# Associations between Childbirth, Hospitalization and Disability Pension: A Cohort Study of Female Twins

**DOI:** 10.1371/journal.pone.0101566

**Published:** 2014-07-07

**Authors:** Emma Björkenstam, Jurgita Narusyte, Kristina Alexanderson, Annina Ropponen, Linnea Kjeldgård, Pia Svedberg

**Affiliations:** 1 Department of Clinical Neuroscience, Division of Insurance Medicine, Karolinska Institutet, Stockholm, Sweden; 2 Finnish Institute of Occupational Health, Helsinki, Finland; Oslo University Hospital, Ullevål, Norway

## Abstract

**Background:**

As the literature on long-term effects of childbirth on risk of morbidity or permanent work incapacity (DP) is limited, we aimed to study associations of childbirth with hospitalization and DP, adjusting for familial factors.

**Methods:**

This cohort study included female twins, i.e. women with twin sister, born 1959–1990 in Sweden (n = 5 118). At least one in the twin pair had their first childbirth 1994–2009. Women were followed regarding all-cause and cause-specific (mental or musculoskeletal diagnoses) DP during year 2–5 after first delivery or equivalent. Associations between childbirth, hospitalization and DP were calculated as hazard ratios (HR) with 95% confidence intervals (CI).

**Results:**

Women who did not give birth had markedly higher number of DP days/year compared to those giving birth. Hospitalization after first childbirth was associated with a higher HR of DP. Those hospitalized at least once after their first childbirth had a three-fold DP risk (HR: 3.2; 95% CI 1.1–9.6), DP due to mental diagnoses (HR: 3.2; 1.2–8.8), and of DP due to musculoskeletal diagnoses (HR: 6.1; 1.6–22.9). Lower HRs in the discordant twin pair analyses indicated that familial factors may influence the studied associations.

**Conclusions:**

Women who did not give birth had a much higher risk for DP than those who did. Among those who gave birth, the risk for DP was markedly higher among those with a previous hospitalization, and especially in women with repeated hospitalizations. The results indicate a health selection into giving birth as well as the importance of morbidity for DP.

## Introduction

Long-term work incapacity in terms of disability pension (DP) is considered a major public health problem with possible severe consequences for individuals, employers, and society [1]. During the last decades, the rate of people on DP has increased in many countries [2].

In most countries with high labor force participation, women have higher DP rates than men [3]–[5]. This gender difference is not fully understood and pregnancy has been suspected to be one of the factors behind this gap [6]. A recent Swedish study showed higher risk for permanent work incapacity for mothers compared to women without children [7]. Although having a child can be considered a positive life event, morbidity related to pregnancy, childbirth or postpartum period might have a significant effect on women's future physical and mental health and work capacity [8]–[11] also in a long-term perspective. It may also be that some women do not give birth due to health-related issues, sometimes referred to as health selection [12]. The literature on long-term effects of childbirth on the risk of morbidity or permanent work incapacity (DP) is very limited, most studies only cover up to a year after delivery.

Many chronic or recurring diseases have a moderate to high heritability [13]–[17]. Individual differences in DP can thus be explained by familial factors (i.e. genetic and shared environment, e.g. during childhood) [18]–[20]. In these studies, about 30–50% of the variance in DP was explained by genetic factors. With use of a twin study design, it is possible to take familial influences into account in the examination of DP in relation to giving birth. Familial factors are those that contribute to the similarity of siblings in a twin pair. So far, little is known about the impact of familial factors on various associations between predictors of DP [18]–[21].

At times, the ill-health content of DPs is questioned [1]. In this study we will study two types of outcomes, morbidity, measured as hospitalization (that is, more severe morbidity) and social consequences of morbidity in terms of long-term or permanent work incapacity due to disease or injury, in terms of DP (in some countries called pension on medical grounds or incapacity benefits) and possible associations between morbidity and DP.

The aim of this study was to investigate the associations of childbirth with hospitalization and DP (all-cause as well as cause-specific) in a cohort of Swedish twins. A secondary aim was to examine if familial factors may be of importance for such associations.

## Methods

### Participants and data sources

**Figure 1:**
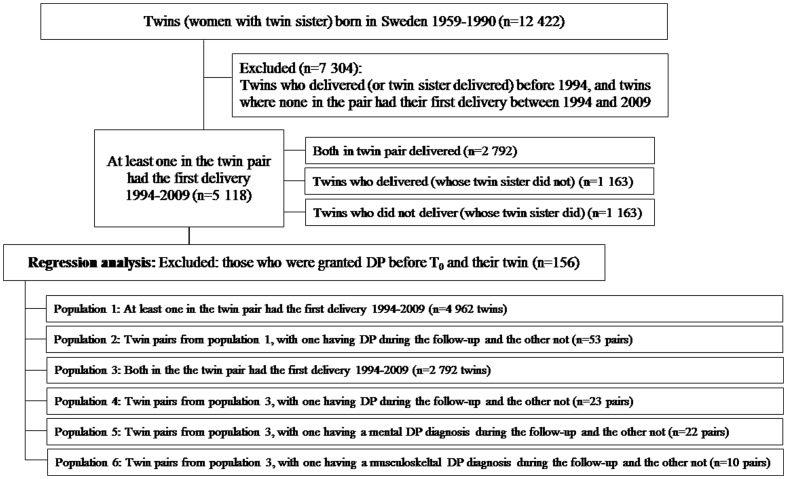
Flow chart for the study population from the Swedish Twin Register.

A prospective population-based cohort study was conducted. The study population was defined as all female twins, i.e. women with a twin sister, born between 1959 and 1990 in Sweden, and recorded in the Swedish Twin Registry (STR) [22]. The STR is the largest population-based register of twin births in the world, with information such as birth date, sex, zygosity, and pair identification [23], [24]. After excluding women who delivered their first child before 1994 (or women whose twin sister had her first delivery before 1994) or before the age of 16, and twins where none in the pair had their first delivery between 1994 and 2009 (n = 7 304), the final cohort comprised 5 118 women. The selection of the study population is illustrated in Figure 1.

Swedish nationwide registers make it possible to perform linkage of data between different registers on an individual level. The unique personal identity number assigned to each Swedish resident [25] was used to link information from several population-based registers. The Causes of Death Register was used to obtain information on death date [26]. The Medical Birth Register, established in 1973, includes information on almost all births in Sweden [27] and was used to obtain information on deliveries from 1994 until 2010. In order to increase coverage on deliveries, we also used the National Patient Register (NPR) to obtain information on delivery. This register includes all individuals admitted to any psychiatric or general hospital since 1987 [28]. Information on hospitalization with a principal diagnosis for delivery (as defined by the International Classification of Disease (ICD) [29]: O80-84) was obtained. The NPR was further used to obtain information on other hospitalizations.

From Statistics Sweden, we obtained information on educational level and year of emigration [30]. Finally, information on DP (date and diagnoses) for the years 1994 to 2009 was obtained from the Social Insurance Agency. DP diagnoses were based on the 9^th^ and 10^th^ revisions of the ICD.

### Time in relation to childbirth

The studied women were followed in relation to year of first child birth, irrespective of birth outcome, referred to as T_0_. In order to compare those who gave birth to those who did not, T_0_ for the women who did not give birth was defined as the year of the twin sister's first childbirth.

### Disability pension and social insurance in Sweden

All residents in Sweden aged 16–64 years are covered by the public sickness insurance and can be granted disability pension (DP), if their work capacity is reduced permanently or for long time due to disease or injury. The DP benefit covers at least 65% of lost income. In Sweden, the customary age of old-age retirement is at 65 years.

### Variables

Hospitalization in terms of inpatient care was studied during the period six years prior to, and six years after T_0_. In a descriptive part, we summarized the total number of hospitalization days per year. In the regression analyses, exposure to hospitalization after T_0_ was considered. In order to consider subsequent pregnancies, we excluded future hospitalizations related to pregnancy, childbirth, and the puerperium (ICD-10: O80-84).

In the descriptive part of the study, total number of DP days each year during the period six years prior to, and six years after T_0_ was summarized. In the analyses on cause-specific DP, diagnoses were classified according to the ICD-9 and ICD-10 at chapter levels for the two diagnostic groups mental disorders (ICD-9: 290–319; ICD-10: F00–F99) and musculoskeletal disorders (ICD-9: 710–739; ICD-10: M00–M99). Also, all participants were followed from year 2–5 after T_0_ with respect to being granted DP.

### Co-twin design

Depending on the zygosity, twins in a pair may share either 100% (monozygotic, MZ) or on average 50% (dizygotic, DZ) of their common genetic make-up. In addition to a similarity depending on the common genes, twins may also become more alike owing to the environment they experience together, i.e. shared (mainly childhood) environment. Co-twin design enables the possibility to take both genetic and shared environmental (also called familial) factors into account by investigating twin pairs discordant for factors of interest. In the discordant twin pair analyses, the follow-up time of the twin mother (on DP) in relation to the follow-up time of the co-twin without DP will be analyzed. Stratification by twin pairs allows each twin pair to have their own baseline hazard to control for the effects of potentially confounding familial factors in the models. That would control for familial factors, meaning that the observed associations found between child birth and hospitalization or DP would not be explained by familial factors shared by the twin sisters in a pair. If the familial factors would play a role, then the association should be present only in the analyses of the whole cohort but not between discordant twin sisters.

### Statistical analysis

Descriptive analyses, based on mean number of days per year in hospital or on DP, were calculated. Cox proportional hazard models with constant time-at-risk [31] were applied to estimate hazard ratios (HR) with 95% confidence intervals (CI) for being granted DP during year 2–5 after T_0_. In the regression analyses, we excluded individuals who were granted DP before T_0_ and their twin (n = 156). The different sub populations in the regression analyses are described in Figure 1. When analyzing the association between childbirth, hospitalization after T_0_ and DP, based on 4 962 twins (referred to as Population 1), three regression models were fitted, where we in Model a only adjusted for birth year. In Model b we made additional adjustments for delivery year and educational level at T_0_. Educational level was classified into four categories; up through 9 years of compulsory school, 10–12 years of education (equivalent to senior high school), ≥13 years of education (i.e. college or university education), and one additional group for missing. In Model c finally, we also adjusted for previous hospitalization, i.e. inpatient care prior to T_0_. Potential familial confounding was controlled for by analyzing twin pairs that were discordant with respect to being granted DP during the follow-up. Conditional Cox proportional models were applied and the sample was stratified by twin pairs [32]. Influence of familial factors (genetic and shared environment) will be indicated if the association found in the analyses of the whole cohort will disappear or change considerably in the analyses of discordant twin pairs. These co-twin analyses included 1 378 complete twin pairs (referred to as Population 3). In order to account for the sampled twin pairs rather than independent individuals, all analyses were clustered on twin-pair identity [33].

Similarly, we estimated HR with 95% CI for the associations between hospitalizations prior to T_0_, after T_0_, and DP, respectively. In these analyses, above described the regression models a and b were used.

All statistical analyses were performed with SAS 9.3 and STATA 12.1.

## Results

Cohort characteristics for the 5 118 individuals are presented in Table 1. Nearly 80% of the women had had one or more childbirths in the studied years 1994 through 2009. Of these women, 71% had a twin sister that also had given birth during these years. Nearly 40% were dizygotic. Most women had an educational level corresponding to high school. A majority of the women who gave birth had been hospitalized some time during the period six years prior to and six years after T_0_ (the delivery year excluded). When excluding hospitalizations with diagnoses for pregnancy and childbirth, approximately 40% of these women had been hospitalized. Among the women who gave birth, 2% were granted DP during the period six years prior to and six years after first delivery, compared to 6% of the women who did not give birth.

**Table 1:** Cohort characteristics for twins (women with twin sister) born in Sweden 1959–1990 (excluding those who delivered before age 16 and those who died before age 16), where at least one in the pair had their first delivery 1994–2009 and none before 1994.

Variables	Both delivered	Twin pairs where only one in the pair delivered		Total
		Twin 1 (delivered)	Twin 2 (not delivered)	
N	2 792	1 163	1 163	5 118
**Number of deliveries**				
One delivery	711 (25%)	501 (43%)		
Two or more deliveries	2 081 (75%)	662 (57%)		
**Age at T_0_**				
16–19 years	47 (2%)	33 (21%)		
20–24 years	511 (18%)	239 (21%)		
25–29 years	1 150 (41%)	444 (38%)		
30–34 years	847 (30%)	323 (28%)		
35–39 years	207 (7%)	109 (9%)		
<40 years	30 (1%)	15 (1%)		
**Zygosity**				
Monozygotic	1 726 (62%)	608 (52%)	608 (52%)	2 942 (57%)
Dizygotic	1 066 (38%)	555 (48%)	555 (48%)	2 176 (43%)
**Highest attained education at T_0_** 1				
≤9 year	179 (6%)	74 (6%)	55 (5%)	308 (6%)
10–12 year	1 434 (51%)	608 (52%)	621 (53%)	2 663 (52%)
≥13 year	1 173 (42%)	474 (41%)	422 (36%)	2 069 (40%)
Information on education missing	6 (0%)	7 (1%)	65 (6%)	78 (2%)
**Hospitalization** 2				
At least one during the period six years prior to and six years after T_0_	2 161 (77%)	703 (60%)	325 (28%)	3 189 (62%)
At least one during the period six years prior to and six years after T_0_ (excluding those with a diagnosis for pregnancy, childbirth, and the puerperium)	865 (31%)	315 (27%)	314 (27%)	1 494 (29%)
At least one during the period six years prior to and six years after T_0_ (excluding those with a diagnosis for pregnancy, and childbirth)	1 183 (42%)	407 (35%)	325 (28%)	1 915 (37%)
At least one during the period six years prior to T_0_	679 (24%)	251 (22%)	217 (19%)	1 147 (22%)
At least one during the period six years after T_0_	1 965 (70%)	585 (50%)	168 (14%)	2 718 (53%)
At least one both before and afterT_0_	483 (17%)	133 (11%)	60 (5%)	676 (13%)
**Disability pension**				
DP during the period six years prior to and six years after T_0_	48 (2%)	33 (3%)	74 (6%)	155 (3%)
DP during the period six years prior to T_0_	18 (1%)	21 (2%)	44 (4%)	83 (2%)
DP during the period six years after T_0_	44 (2%)	25 (2%)	59 (5%)	128 (3%)

T_0_ is defined as year for first childbirth for women who gave birth, and year of twin sister's first childbirth for those who did not give birth.

1 At T_0_

2 Excluding hospitalizations occurring during T_0_

**Figure 2:**
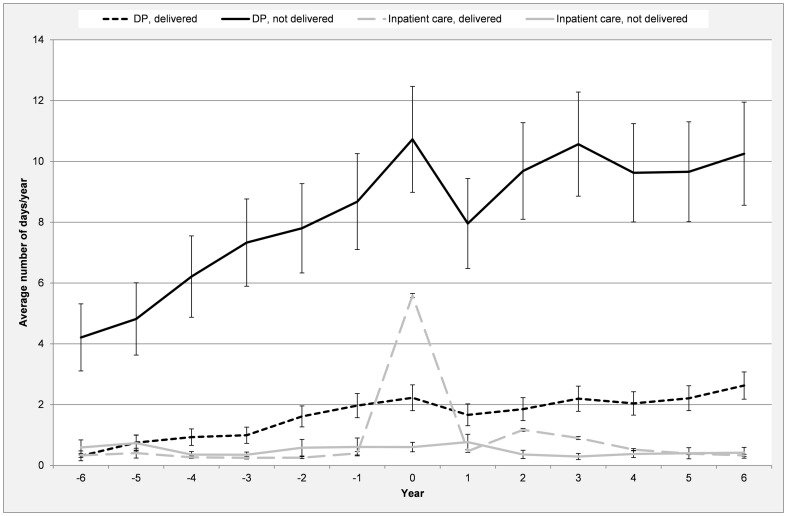
Average number of DP and inpatient care days, respectively, per year (with 95% CI), six years prior to and six year after T0 for women who delivered/not delivered (n = 5 118).

The women who did not give birth had markedly higher average number of DP days/year compared to those who gave birth (Figure 2). The average number of days on DP steadily increased over the studied years for both groups. The annual average number of hospitalization days increased substantially during the year for the first childbirth and decreased thereafter.

Table 2 presents HR for the association between childbirth, hospitalization and DP. Based on the results from the crude model (model a), women who did not give birth had a higher HR of future DP, especially those who had been hospitalized at least once during year 1–2 after T_0_ when compared to those who had given birth and not been hospitalized (HR: 26.4; 95% CI 12.9–54.1). The higher risk remained in the fully adjusted model (model c), however, with lower, but still very high estimates (HR: 17.0; 95% CI 8.1–35.9). The analyses of twin pairs discordant for receiving DP year 2–5 after T_0,_ show attenuated point estimates, hence suggesting that familial effects play a role for the associations.

**Table 2:** Cox proportional HR with 95% CI for the association between childbirth, hospitalization and disability pension (DP) during year 2–5 after T_0_, in twins where at least one in the pair had their first delivery 1994–2009, and none before 1994.

Status childbirth and hospitalization	DP			
	Model a^3^	Model b^4^	Model c^5^	Population 2^1^^,^ 5
**All women (n = 4 962)**	**DP year 2**–**5**			
Childbirth	1 (REF)	1 (REF)	1 (REF)	1 (REF)
No childbirth	3.0 (1.8–4.9)	3.4 (2.0–5.8)	3.6 (2.1–6.0)	2.0 (1.1–3.5)
Childbirth, no hospitalization year 1–2 after T_0_ 6	1 (REF)	1 (REF)	1 (REF)	1 (REF)
Childbirth, hospitalization year 1–2 after T_0_ 6	3.4 (1.2–9.6)	2.6 (0.9–7.6)	2.4 (1.3–4.5)	1.7 (0.9–3.3)
No childbirth, no hospitalization year 1–2 after T_0_ 6	2.1 (1.1–3.8)	2.4 (1.3–4.4)	2.4 (1.3–4.5)	1.7 (0.9–3.3)
No childbirth, hospitalization year 1–2 after T_0_ 6	26.4 (12.9–54.1)	22.6 (11.0–46.6)	17.0 (8.1–35.9)	2.5 (1.2–5.5)
	**Model a** 3	**Model b** 4	**Model c** 5	**Population 4** 2 **^,^** 5
**Women who had at least one delivery (n = 2 756)**				
No hospitalization year 1–2 after T_0_ 6	1 (REF)	1 (REF)	1 (REF)	1 (REF)
Hospitalization year 1–2 after T_0_ 6	4.5 (1.5–13.0)	3.4 (1.2–10.0)	3.2 (1.1–9.6)	0.9 (0.3–2.6)

T_0_ is defined as year for first childbirth for women who gave birth, and year of twin sister's first childbirth for those who did not give birth.

1 Twin pairs with one having DP during the follow-up and the other not (n = 53 pairs).

2 Twin pairs (both giving birth) with one having DP during the follow-up and the other not (n = 23 pairs).

3 Adjusted for birth year.

4 Model a, with additional adjustments for delivery year and educational level.

5 Model b, with additional adjustments for earlier hospitalization.

6 Diagnoses related to pregnancy, childbirth and the puerperium excluded (ICD-10: O80-84).

When restricting the analyses to those who gave birth and excluding those with DP before T_0_, 2 756 mothers were included (Table 2). Mothers who had been hospitalized during year 1–2 after their first childbirth had a higher risk of future DP, when compared to those who had not had such hospitalization (HR: 4.5; 95% CI 1.5–13.0). This association remained even when hospitalizations due to subsequent pregnancies were removed. The risk estimates decreased slightly in the fully adjusted model (model c) and disappeared after controlling for familial confounding.

The HRs for diagnosis-specific DP among women giving birth between 1994 and 2009, presented in Table 3, show that mothers hospitalized after first delivery had a higher risk of DP due to musculoskeletal diagnoses (HR: 6.0; 95% CI 1.6–22.5). These women also had a higher risk for being granted DP due to mental diagnoses (HR: 3.2; 95% CI 1.2–8.8 in the fully adjusted model).

**Table 3:** Cox proportional HR with 95% CI for the association between childbirth, hospitalization and DP due to mental or musculoskeletal diagnoses during year 2–5 after first childbirth, in twins, where at least one in the pair had their first delivery 1994–2009 and none before 1994.

Status childbirth and hospitalization	Mental DP diagnosis				Musculoskeletal DP diagnosis			
	Model a^3^	Model b^4^	Model c^5^	Discordant twin pairs^5^	Model a^3^	Model b^4^	Model c^5^	Discordant twin pairs^5^
**All women (n = 4 962)**								
Childbirth	1 (REF)	1 (REF)	1 (REF)	1 (REF)	1 (REF)	1 (REF)	1 (REF)	1 (REF)
No childbirth	2.5 (1.5–4.3)	3.3 (1.9–5.7)	3.4 (2.0–5.9)	2.5 (1.3–4.6)	0.85 (0.2–3.0)	1.0 (0.3–3.6)	1.0 (0.3–3.6)	2.4 (0.6–10.6)
**Women who had at least one delivery (n = 2 756)**	**Model a** 3	**Model b** 4	**Model c** 5	**Population 5** 1 **^,^** 5	**Model a** 3	**Model b** 4	**Model c** 5	**Population 6** 2 **^,^** 5
No hospitalization year 1–2 after T_0_ 6	1 (REF)	1 (REF)	1 (REF)	1 (REF)	1 (REF)	1 (REF)	1 (REF)	1 (REF)
Hospitalization year 1–2 after T_0_ 6	5.1 (1.9–13.4)	3.7 (1.4–9.8)	3.2 (1.2–8.8)	1.3 (0.4–3.8)	6.0 (1.6–22.5)	6.0 (1.6–22.5)	6.1 (1.6–22.9)	1.5 (0.2–8.8)

T_0_ is defined as year for first childbirth for women who gave birth, and year of twin sister's childbirth for those who did not give birth.

1 Twin pairs with one twin having a mental DP diagnosis during the follow-up and the other not (n = 22 pairs).

2 Twin pairs with one twin having a musculoskeletal DP diagnosis during the follow-up and the other not (n = 10 pairs).

3 Adjusted for birth year.

4 Model a, with additional adjustments for delivery year and educational level.

5 Model b, with additional adjustments for earlier hospitalization.

6 Diagnoses related to pregnancy, childbirth and the puerperium excluded (ICD-10: O80-84).

The analyses of hospitalization before and after first childbirth and future DP (Table 4), show that hospitalization during year 1–2 after T_0_ was the strongest predictor for future DP (HR: 5.4; 95% CI 1.6–18.9). Compared to mothers who were neither hospitalized before nor after first childbirth, a slight risk increase was also observed for those who were hospitalized before T_0_. In the analyses of twin pairs discordant for being granted DP year 2–5 after childbirth, the HRs were reduced to 0.7–1.0, suggesting that the associations were explained by familial effects.

**Table 4:** Cox proportional HR with 95% CI for the association between hospitalization before and after first childbirth, and DP during year 2–5 after first childbirth, in twins who had their first delivery 1994–2009 (n = 2 756).

Status hospitalization before and after childbirth	DP (any diagnosis)		
	Model a^4^	Model b^5^	Population 4^1^^,^ 5
No hospitalization either before T_0_ 6 or during year 1–2 after T_0_ 7	1 (REF)	1 (REF)	1 (REF)
Hospitalization before T_0_ 6 but not during year 1–2 after T_0_ 7	2.2 (0.8–5.7)	1.9 (0.7–4.9)	1.1 (0.4–3.2)
No hospitalization before T_0_ 6 but during year 1–2 after T_0_ 7	5.4 (1.6–18.9)	4.3 (1.2–15.0)	1.0 (0.3–3.8)
Hospitalization before T_0_ 6 and during year 1–2 after T_0_ 7	5.1 (0.7–38.7)	3.3 (0.4–25.4)	0.7 (0.1–6.0)
	**Mental DP diagnosis**		
	**Model a** 4	**Model b** 5	**Population 5** 2 **^,^** 5
No hospitalization either before T_0_ 6 or during year 1–2 after T_0_ 7	1 (REF)	1 (REF)	1 (REF)
Hospitalization before T_0_ 6 but not during year 1–2 after T_0_ 7	2.9 (1.2–7.0)	2.5 (1.0–6.1)	0.7 (0.3–2.1)
No hospitalization before T_0_ 6 but during year 1–2 after T_0_ 7	5.4 (1.6–18.9)	4.3 (1.2–15.1)	1.0 (0.3–3.8)
Hospitalization before T_0_ 6 and during year 1–2 after T_0_ 7	10.1 (2.3–44.3)	5.8 (1.3–26.1)	1.6 (0.3–7.7)
	**Musculoskeletal DP diagnosis**		
	**Model a** 4	**Model b** 5	**Population 6** 3 **^,^** 5
No hospitalization either before T_0_ 6 or during year 1–2 after T_0_ 7	1 (REF)	1 (REF)	1 (REF)
Hospitalization before T_0_ 6 but not during year 1–2 after T_0_ 7	0.6 (0.1–5.1)	0.6 (0.1–4.7)	1.1 (0.1–11.5)
No hospitalization before T_0_ 6 but during year 1–2 after T_0_ 7	6.3 (1.3–29.7)	4.9 (1.0–23.6)	2.3 (0.2–26.8)
Hospitalization before T_0_ 6 and during year 1–2 after T_0_ 7	9.0 (1.1–72.0)	7.3 (0.9–60.2)	1.1 (0.1–10.8)

1 Twin pairs with one twin having DP during the follow-up and the other not (n = 23 pairs).

2 Twin pairs with one twin having a mental DP diagnosis during the follow-up and the other not (n = 22 pairs).

3 Twin pairs with one twin having a musculoskeletal DP diagnosis during the follow-up and the other not (n = 10 pairs).

4 Adjusted for birth year.

5 Model a, with additional adjustments for delivery year, and educational level.

6 During the period 1–5 years prior to T0.

7 Diagnoses related to pregnancy, childbirth and the puerperium excluded (ICD-10: O80-84).

The strongest predictor for being granted DP with mental diagnoses was being hospitalized both before and after first childbirth (HR: 10.1; 95% CI 2.3–44.3). The HR decreased slightly in the fully adjusted model. When we controlled for familial confounding, the HR was also reduced, suggesting that familial factors may influence the studied association.

Being hospitalized both before and after T_0_ was also a strong predictor for being granted DP with musculoskeletal diagnoses (HR: 9.0; 95% CI 1.1–72.0). In the discordant twin pair analysis, HRs were reduced, indicating that familial factors seemed to influence this association.

## Discussion

Our prospective cohort study of 5 118 female twins in Sweden, in which we studied the association between childbirth, hospitalization and DP, showed elevated risks for future DP in women who did not give birth. Moreover, hospitalization was a prominent risk factor for subsequent DP, regardless of childbirth status. Familial factors played a role in the studied association.

### Interpretation and comparison with other studies

To our knowledge, this is the first study to examine the long-term associations between childbirth, morbidity and DP. One recent study analyzing the influence of low social interaction and socioeconomic risk conditions on the risk of DP in a cohort of all Swedish women born in 1960 to 1979 and not on DP, found that women not living with children and especially women living alone, had higher risk for DP when compared to women with children [7]. Similar findings were reported in another Swedish study of the same cohort and the DP risks were still lower among those having three or more children [34]. In some of the analyses, women with children had higher risk of future DP [34]. Worth noting, however, is that these two studies focused on motherhood rather than on giving birth and that also women not born in Sweden were included.

Our results also revealed that women who did not give birth had higher risk for DP due to mental diagnoses; this has not been studied before. An Icelandic study on DP due to mental disorders pointed out single mothers with poor financial status to be of greater risk of mental disorder that can lead to DP [35]. There are also findings suggesting that childlessness is not linked to mental disorder [36], [37]. However, these two studies did not use DP diagnoses but rather self-reported information on disorders [36], [37]. It is of course important to be aware of that most women, in both groups, were not granted DP at all.

In general, women who did not give birth had a higher risk for future DP. One could expect that a health selection, where some of the women who do not give birth may have worse health and therefore cannot get pregnant or choose not to have a child. However, several women choose not to have children due to other reasons. Studies comparing death rates in pregnant women with death rates in other women have showed a health selection possibly combined with an apparent protective effect of pregnancy on women's health [38]–[40]. Women who have given birth may thus have a greater proportion of healthy women.

The analyses of risk for DP due to musculoskeletal diagnoses showed the opposite, i.e. that women who gave birth had a higher risk for such DP. This is in line with previous research, showing that childbirth may be associated with higher risk for future musculoskeletal disorders [10], [11].

The risk of future DP was especially elevated in women who had experienced a hospitalization the years following the first childbirth or equivalent. Hospitalization, that is, our measure of morbidity, remained a significant predictor for being granted DP even when previous hospitalization, i.e. inpatient care prior to T_0_, was accounted for. As it is known that low socioeconomic position is a risk factor for being granted DP [7], [41], we adjusted for educational level. However, this adjustment barely affected the estimates, leading to the conclusion that morbidity in terms of hospitalization is a strong risk factor for future DP. This might seem self-evident, nevertheless, often questioned in public discussions of the ill-health content of DPs. Previous research on the association between hospitalization and subsequent DP is scarce, and even though studies have shown that morbidity is associated with DP, most studies have focused on self-rated health and self-reported conditions rather than hospitalization as an indicator of morbidity [7], [34], [42], [43]. Also, repeated hospitalization, which could be regarded as a more severe measure of morbidity, was associated with an elevated risk for future DP.

Familial factors seemed to influence the association between childbirth and DP, between hospitalization and DP for those who gave birth, and also between repeated hospitalizations and DP. One possible explanation could be that more severe morbidity implies more severe disorders, and pathology generally shows higher heritability estimated than symptoms. For example, heritability of mental disorders varies between 28–85% [17], with higher estimates for major depression and bipolar disorder vs depressed mood [44], whereas genetic effects explained approximately 30–60% [13], [14] of the variation in musculoskeletal disorders.

### Strengths and limitations

The strengths of this study include the population-based prospective design, using nationwide registers with high completeness and no loss to follow up, and the large cohort. The register-based data counteracts recall or selection bias. The National Patient Register has close to complete coverage of all inpatient care [28]. Other strengths include the use of a co-twin design, which enables to take into account the influence of familial factors, the prospective design with a long follow-up period, and that also information on the situation before T_0_ could be included. That we had access to information about DP diagnoses was another strength.

There are, however, some weaknesses that should be mentioned. First of all, the terms for inpatient care have changed in Sweden during the studied period, and inpatient care has to a large extent been replaced by outpatient treatment, thus the rates of hospitalization have steadily decreased. Hence, in our study, some patients who in the later studied years were treated in outpatient care are classified as unexposed, as there is no record of their outpatient visit. However, the impact of this would be the same for those giving and not giving birth. Another limitation was that there were relatively few twin pairs who were discordant with respect to DP. Precision was relatively low, as indicated by the wide CIs. Hence, the results of the discordant twin pair analyses for DP should be interpreted with caution. Moreover, it is important to remember that the results concern women who gave birth, irrespective if the child survived or not or if the woman lived with the child. Other studies include information on being a mother, and might also include women who live with children she did not give birth to.

## Conclusion

In conclusion, our study shows a marked higher risk for DP in women who did not give birth compared to those who did. Also, previous hospitalization (e.g. morbidity) was a risk factor for future DP, both in women who did or did not give birth. This was especially evident in women with repeated hospitalizations. Repeated hospitalizations also seemed to play a role that was confirmed in the analyses controlling for familial effects. Thus, it seems that familial factors should be accounted for when investigating the associations between hospitalizations and DP after childbirth. The findings also suggest health selection into not giving birth.

### Ethical approval

The study was approved by the Regional Ethical Review Board of Karolinska Institutet, Stockholm, Sweden (2007/524-31).

## References

[1] Alexanderson K, Norlund A (2004) Swedish Council on Technology Assessment in Health Care (SBU). Chapter 1. Aim, background, key concepts, regulations, and current statistics. Scandinavian Journal of Public Health 32: 12–30. 10.1080/14034950410021808 15513650

[2] OECD (2009) Sickness, Disability and Work: Breaking the Barriers, Sweden - Will the recent reforms make it?

[3] Borg K, Hensing G, Alexanderson K (2004) Risk factors for disability pension over 11 years in a cohort of young persons initially sick-listed with low back, neck, or shoulder diagnoses. Scand J Public Health 32: 272–278. 15370767 10.1080/14034940310019524

[4] Haukenes I, Gjesdal S, Rortveit G, Riise T, Maeland JG (2012) Women's higher likelihood of disability pension: the role of health, family and work. A 5–7 years follow-up of the Hordaland Health Study. BMC Public Health 12: 720. 22943493 10.1186/1471-2458-12-720 PMC3508825

[5] Mykletun A, Overland S, Dahl AA, Krokstad S, Bjerkeset O, et al (2006) A population-based cohort study of the effect of common mental disorders on disability pension awards. Am J Psychiatry 163: 1412–1418. 16877655 10.1176/ajp.2006.163.8.1412

[6] Vistnes JP (1997) Gender differences in days lost from work due to illness. Industrial & Labor Relations Review 50: 304–323.

[7] Gustafsson K, Aronsson G, Marklund S, Wikman A, Hagman M, et al.. (2013) Social Integration, Socioeconomic Conditions and Type of Ill Health Preceding Disability Pension in Young Women: a Swedish Population-Based Study. Int J Behav Med. 10.1007/s12529-012-9287-5 23307701

[8] Cheng C, Li Q (2008) Integrative review of research on general health status and prevalence of common physical health conditions of women after childbirth. Womens Health Issues 18: 267–280. 18468922 10.1016/j.whi.2008.02.004

[9] Schytt E, Lindmark G, Waldenstrom U (2005) Physical symptoms after childbirth: prevalence and associations with self-rated health. BJOG An International Journal of Obstetrics and Gynaecology 112: 210–217. 15663586 10.1111/j.1471-0528.2004.00319.x

[10] Borg-Stein J, Dugan SA (2007) Musculoskeletal disorders of pregnancy, delivery and postpartum. Phys Med Rehabil Clin N Am 18: : 459–476, ix. 10.1016/j.pmr.2007.05.005 17678762

[11] To WW, Wong MW (2011) Persistence of back pain symptoms after pregnancy and bone mineral density changes as measured by quantitative ultrasound—a two year longitudinal follow up study. BMC Musculoskelet Disord 12: 55. 21352600 10.1186/1471-2474-12-55 PMC3053307

[12] Beckmann CRB, Ling FW, Barzansky BM, Herbert WNP, Laube DW, et al.. (2010) Obstetrics and gynecology: Lippincott Williams & Wilkins.

[13] Battie MC, Videman T, Levalahti E, Gill K, Kaprio J (2007) Heritability of low back pain and the role of disc degeneration. Pain 131: 272–280. 17335977 10.1016/j.pain.2007.01.010

[14] MacGregor AJ, Snieder H, Rigby AS, Koskenvuo M, Kaprio J, et al (2000) Characterizing the quantitative genetic contribution to rheumatoid arthritis using data from twins. Arthritis Rheum 43: 30–37. 10643697 10.1002/1529-0131(200001)43:1<30::AID-ANR5>3.0.CO;2-B

[15] Plomin R, DeFries J, McClean GE, McGuffin P (2000) Behavioral Genetics. New York: Worth Publishers.

[16] Plomin R, Owen MJ, McGuffin P (1994) The genetic basis of complex human behaviors. Science 264: 1733–1739. 8209254 10.1126/science.8209254

[17] Bienvenu OJ, Davydow DS, Kendler KS (2011) Psychiatric ‘diseases’ versus behavioral disorders and degree of genetic influence. Psychol Med 41: 33–40. 20459884 10.1017/S003329171000084X PMC10885735

[18] Gjerde LC, Knudsen GP, Czajkowski N, Gillespie N, Aggen SH, et al (2013) Genetic and environmental contributions to long-term sick leave and disability pension: a population-based study of young adult Norwegian twins. Twin Res Hum Genet 16: 759–766. 23743022 10.1017/thg.2013.36 PMC3800163

[19] Harkonmaki K, Silventoinen K, Levalahti E, Pitkaniemi J, Huunan-Seppala A, et al (2008) The genetic liability to disability retirement: a 30-year follow-up study of 24,000 Finnish twins. PLoS One 3: e3402. 18923678 10.1371/journal.pone.0003402 PMC2566596

[20] Narusyte J, Ropponen A, Silventoinen K, Alexanderson K, Kaprio J, et al. (2011) Genetic liability to disability pension in women and men: a prospective population-based twin study. PLoS One 6.. 10.1371/journal.pone.0023143 PMC3151284 21850258

[21] Ropponen A, Svedberg P (2013) Single and additive effects of health behaviours on the risk for disability pensions among Swedish twins. Eur J Public Health. 10.1093/eurpub/ckt168 24196487

[22] Lichtenstein P, De Faire U, Floderus B, Svartengren M, Svedberg P, et al (2002) The Swedish Twin Registry: a unique resource for clinical, epidemiological and genetic studies. J Intern Med 252: 184–205. 12270000 10.1046/j.1365-2796.2002.01032.x

[23] Furberg H, Lichtenstein P, Pedersen NL, Thornton L, Bulik CM, et al (2008) The STAGE cohort: a prospective study of tobacco use among Swedish twins. Nicotine Tob Res 10: 1727–1735. 18988069 10.1080/14622200802443551 PMC2914543

[24] Lichtenstein P, Sullivan PF, Cnattingius S, Gatz M, Johansson S, et al (2006) The Swedish Twin Registry in the third millennium: an update. Twin Res Hum Genet 9: 875–882. 17254424 10.1375/183242706779462444

[25] Ludvigsson JF, Otterblad-Olausson P, Pettersson BU, Ekbom A (2009) The Swedish personal identity number: possibilities and pitfalls in healthcare and medical research. Eur J Epidemiol 24: 659–667. 19504049 10.1007/s10654-009-9350-y PMC2773709

[26] National Board of Health and Welfare (2011) Causes of Death 2010. Stockholm, Sweden.

[27] Cnattingius S, Ericson A, Gunnarskog J, Kallen B (1990) A quality study of a medical birth registry. Scand J Soc Med 18: 143–148. 2367825 10.1177/140349489001800209

[28] Ludvigsson JF, Andersson E, Ekbom A, Feychting M, Kim JL, et al (2011) External review and validation of the Swedish national inpatient register. BMC Public Health 11: 450. 21658213 10.1186/1471-2458-11-450 PMC3142234

[29] World Health Organization (1992) ICD 10: International Statistical Classification of Diseases and Related Health Problems. Geneva, Switzerland.

[30] Statistics Sweden (2012) Longitudinal integration database for health insurance and labour market studies (LISA by Swedish acronym).

[31] Barros AJ, Hirakata VN (2003) Alternatives for logistic regression in cross-sectional studies: an empirical comparison of models that directly estimate the prevalence ratio. BMC Med Res Methodol 3: 21. 14567763 10.1186/1471-2288-3-21 PMC521200

[32] Kujala UM, Kaprio J, Koskenvuo M (2002) Modifiable risk factors as predictors of all-cause mortality: the roles of genetics and childhood environment. Am J Epidemiol 156: 985–993. 12446254 10.1093/aje/kwf151

[33] Williams D (2003) Pregnancy: a stress test for life. Current opinion in obstetrics & gynecology 15: 465–471. 14624211 10.1097/00001703-200312000-00002

[34] Floderus B, Hagman M, Aronsson G, Gustafsson K, Marklund S, et al. (2012) Disability pension among young women in Sweden, with special emphasis on family structure: a dynamic cohort study. BMJ Open 2.. 10.1136/bmjopen-2012-000840 PMC3367147 22649174

[35] Thorlacius S, Stefansson SB, Olafsson S, Tomasson K (2010) Increased incidence of disability due to mental and behavioural disorders in Iceland 1990–2007. J Ment Health 19: 176–183. 20433325 10.3109/09638230902968316

[36] Cwikel J, Gramotnev H, Lee C (2006) Never-married childless women in Australia: health and social circumstances in older age. Soc Sci Med 62: 1991–2001. 16225976 10.1016/j.socscimed.2005.09.006

[37] Koropeckyj-Cox T, Pienta AM, Brown TH (2007) Women of the 1950s and the “normative” life course: the implications of childlessness, fertility timing, and marital status for psychological well-being in late midlife. Int J Aging Hum Dev 64: 299–330. 17703677 10.2190/8PTL-P745-58U1-3330

[38] Ronsmans C, Khlat M, Kodio B, Ba M, De Bernis L, et al (2001) Evidence for a ‘healthy pregnant woman effect’ in Niakhar, Senegal? Int J Epidemiol 30: 467–473 discussion 474–465. 11416066 10.1093/ije/30.3.467

[39] Gissler M, Kauppila R, Merilainen J, Toukomaa H, Hemminki E (1997) Pregnancy-associated deaths in Finland 1987–1994—definition problems and benefits of record linkage. Acta Obstet Gynecol Scand 76: 651–657. 9292639 10.3109/00016349709024605

[40] Jocums SB, Berg CJ, Entman SS, Mitchell EF Jr (1998) Postdelivery mortality in Tennessee, 1989-1991. Obstet Gynecol 91: 766–770. 9572227 10.1016/s0029-7844(98)00063-5

[41] Allebeck P, Mastekaasa A (2004) Swedish Council on Technology Assessment in Health Care (SBU). Chapter 5. Risk factors for sick leave - general studies. Scand J Public Health Suppl: 49–108. 10.1080/14034950410021853 15513654

[42] Halford C, Wallman T, Welin L, Rosengren A, Bardel A, et al (2012) Effects of self-rated health on sick leave, disability pension, hospital admissions and mortality. A population-based longitudinal study of nearly 15,000 observations among Swedish women and men. BMC Public Health 12: 1103. 23259777 10.1186/1471-2458-12-1103 PMC3607994

[43] Pietikäinen S, Silventoinen K, Svedberg P, Alexanderson K, Huunan-Seppälä A, et al. (2011) Health-Related and Sociodemographic Risk Factors for Disability Pension due to Low Back Disorders: A 30-Year Prospective Finnish Twin Cohort Study. Journal of Occupational and Environmental Medicine 53.. 10.1097/JOM.0b013e31821576dd 21522028

[44] Kendler KS (1995) Genetic epidemiology in psychiatry. Taking both genes and environment seriously. Arch Gen Psychiatry 52: 895–899. 7487337 10.1001/archpsyc.1995.03950230009003

